# Birth Cohort Studies are Necessary to Understand the Epidemiology and Nature of Non-Communicable Diseases in Africa

**DOI:** 10.4314/ejhs.v34i5.1

**Published:** 2024-09

**Authors:** Daniel Yilma, Tsinuel Girma

**Affiliations:** 1 Department of Internal Medicine, Jimma University, Jimma, Ethiopia; 2 Department of Pediatrics and Child Health, Jimma University, Jimma, Ethiopia; 3 Fenot Project, Department of Global Health and Population, Harvard T.H. Chan School, Addis Ababa, Ethiopia

The burden of non-communicable diseases (NCDs) has been increasing over the past decade, with 41 million people dying each year, accounting for 74% of all global deaths. Cardiovascular diseases, cancers, chronic respiratory diseases, and diabetes are the four major groups of diseases that contribute to over 80% of all premature NCD deaths (1). In Africa, over 1.6 million people aged 30 to 70 die prematurely each year from at least one of these four major NCDs. Common risk factors for NCDs include tobacco use, harmful alcohol consumption, unhealthy diet, lack of physical activity, overweight/obesity, high blood pressure, high blood sugar, and high cholesterol (1). However, these risk factors do not fully explain the epidemiology and pathways of NCDs in Africa.

The risk of NCDs is often influenced by complex interactions of developmental, social, and environmental factors. The effects of adverse events during fetal and early life are interconnected with and influenced by later exposures and lifestyle. Birth cohort studies are valuable in understanding the relationship between NCD risk factors during the prenatal or postnatal period and later in life. Several large birth cohort studies conducted in high-income countries have expanded our understanding of NCD risk and led to significant discoveries (2,3). However, the context of early life growth and environmental exposure differ between high and low-income countries, indicating the need for context-specific evidence.

Prospective birth cohort studies that follow mother-infant dyads help collect comprehensive data, including biological samples. However, the cohorts require substantial investment and strong organizational capacities. In Africa, a growing number of birth cohorts have been reported in the last decades, but many experienced challenges such as high rates of attrition and insufficient funding for follow-up (4). Most developed countries have transitioned to population-based birth cohorts by linking birth registries with health administrative databases or electronic health records, allowing the assessment of links between early-life growth and later-life disease risk in large populations (5). Therefore, it is crucial to support existing birth cohort studies in Africa and strengthen the health system, including medical and birth registries, to establish a population-based birth cohort for a better understanding of the increasing burden of NCDs in Africa.
